# Study on the Cytotoxic, Genotoxic and Clastogenic Potential of *Attalea phalerata* Mart. ex Spreng. Oil Pulp *In Vitro* and *In Vivo* Experimental Models

**DOI:** 10.1371/journal.pone.0165258

**Published:** 2016-10-20

**Authors:** Fernando Freitas de Lima, Sara Emilia Lima Tolouei Menegati, Giseli Karenina Traesel, Flávio Henrique Souza de Araújo, Caroline Honaiser Lescano, Sara Moraes Peixoto, Felipe Ariel Mao Silva, Silvia Cristina Heredia Vieira, Maria do Carmo Vieira, Silvia Aparecida Oesterreich

**Affiliations:** 1 Faculty of Health Science, Federal University of Grande Dourados, Dourados, Brazil; 2 Faculty of Biological and Environmental Sciences, Federal University of Grande Dourados, Dourados, Brazil; 3 Faculty of Agricultural Sciences, Federal University of Grande Dourados, Dourados, Brazil; 4 Biodiversity Center, State University of Mato Grosso do Sul, Dourados, Brazil; 5 Center of Biological and Health Sciences, Federal University of Mato Grosso do Sul, Campo Grande, Brazil; 6 Faculty of Medicinal Sciences, State University of Campinas, Campinas, Brazil; Indiana University, UNITED STATES

## Abstract

*Attalea phalerata* Mart. ex Spreng. (*Arecaceae*), popularly known as “bacuri”, is used in Brazilian folk medicine. Its oil is used orally to relieve pulmonary congestion and joint pain. In topical applications, it is applied as an effective hair tonic and anti-dandruff. The *in natura* pulp and its nuts are used as food because of its nutritional value. Despite its use in folk medicine, there is a lack of data regarding its *in vivo/in vitro* cytotoxic/genotoxic and clastogenic effects. Therefore, in this study, we evaluated the cytotoxic, genotoxic and clastogenic effects of *Attalea phalerata* Mart. ex Spreng. oil (APMO) *in vitro* and *in vivo*. For the analysis of cytotoxic potential, the *Artemia salina* and MTT (3-(4,5-dimethizzol-zyl)-2,5-diphenyltetrazolium bromide) assays were performed. Possible cytotoxic, genotoxic and clastogenic effects of APMO intake were determined by performing the comet and micronucleus assays. Male and female *Wistar* rats were orally treated with doses of 125, 250, 500 or 1000 mg.kg^-1^ of the APMO daily for 28 consecutive days (four weeks). The results showed that the APMO did not induce cell death in the experiments of *Artemia salina* and MTT, indicating that it has no cytotoxicity. The APMO did not cause significant damage to the DNA of the rats in the four doses used when compared to the negative control group (saline + Tween^®^ 80). The APMO did not present any significant increase in micronucleated polychromatic erythrocytes (MNPCEs) for the four tested doses. When compared to the positive control group, all groups (comet and micronucleus tests) were statistically different. These data suggest that the administration of *Attalea phalerata* Mart oil. ex Spreng does not cause cytotoxicity, genotoxicity and clastogenicity in experimental models *in vitro* and *in vivo* following oral administration in this study.

## Introduction

The therapeutic use of natural products, including medicinal plants, has become increasingly common. Pharmacological investigations are performed to identify bioactive compounds with beneficial abilities to the human organism in order to develop new drugs with reduced side effects [1, 2]. Due to the biological activity of these compounds, the evaluation of the toxic potential is essential for the safe and effective use of medicinal plants [3, 4]. Besides that, the phytochemical study of plants and foods with medicinal properties are important in order to learn about the natural compounds and their mechanisms of action [5, 6].

*Attalea phalerata* Mart. ex Spreng. (*Arecaceae*) belongs to the genus Attalea Kunth and is popularly known as ''bacuri'' or ''acuri''. In folk medicine, the pulp oil is used as a hair tonic and anti-dandruff. Orally, the oil is applied to relieve pulmonary congestion, joint pain and studies indicate the anti-inflammatory properties of the pulp oil due to its chemical composition [7, 8, 9]. The almond and the fruit pulp are consumed by the local population as they present high nutritional value. Some fruits of the Cerrado have similar chemical constituents, such as carotenoids and fatty acids. These compounds are of paramount importance to the pharmaceutical industry because of its pharmacological potential. Among its activities are the maintenance of the immune system and prevention of chronic diseases [10–12]. Recently, studies with Cerrado fruits have demonstrated pharmacological activities in rats [7, 13, 14].

Early in the development of a pharmaceutical there are a number of preliminary tests that basis for validating the safety of the natural chemical compounds and potentially the development of new pharmaceutical products [15–17]. According to the literature, the *Artemia salina* test is considered one of the most useful tools for preliminary tests assessing general toxicity at low cost and shows good correlation with cytotoxic activity [18, 19]. With the MTT (3-(4,5-dimethizzol-zyl)-2,5-diphenyltetrazolium bromide) assay, it is possible to assess the cytotoxicity and this test is used with great success for estimating the number of viable cells in the initial screening for new drugs [20]. Through the comet assay it is possible to evaluate the genotoxic potential of a substance and the micronucleus assay provides information on cytotoxic and clastogenic effects. The use of both tests jointly is recognized by international regulatory agencies [21, 22], since the assays are very sensitive and detect breaks in the chromosomal and chromatid levels [23]. Therefore, the present study was designed to investigate the cytotoxic, genotoxic and clastogenic potentials of the pulp oil of *Attalea pha*lerata Mart. ex Spreng. in *in vitro* and *in vivo* experimental models.

## Material and Methods

### Material and sample preparation

The *Attalea phalerata* Mart. ex Spreng. fruit was collected from a public area of the municipality of Rio Brilhante—MS, 21° 55' 04.6"S and 54° 32' 06.8"W and altitude 6 m. No specific permissions were required to access the area in which the fruits were collected since it is a public area (highway). The species (*A*. *phalerata* Mart.) used in this study is not an endangered or protected species. The plant name is in accordance with the on-line database published by “The Plant List”, accessed on May 02, 2016. A voucher specimen of the species was deposited in the UFGD DDMS Herbarium under the number 5033. After the collection, the healthy fruits were washed with tap water and immersed in a sanitized solution of sodium dichloroisocyanurate 0.66% (content of active chlorine of 3%) for 10 minutes. Afterwards, the fruits were peeled, pulped and the pulp was subsequently dried in an oven at 40°C with an air flow of 0.5 m.s^-1^ for 72 hours. The dried material was crushed, sieved through a 20-mesh sieve for powder uniformity, subsequently packaged in flexible polyethylene packages and stored at room temperature.

### Oil extraction

The *Attalea phalerata* Mart. ex Spreng. oil (APMO) was obtained by Soxhlet extraction with hexane solvent PA (Vetec) at a ratio of 3 part dewatered pulp powder to 6 parts solvent 3:6 (w.v^-1^) under continuous extraction until sample exhaustion. The product was filtered, the solvent removed and the oil stored in low temperature (3°C) until further analysis.

### Chemicals

For the analysis of carotenoids by high-performance liquid chromatography, β-carotene (≥97% Sigma-Aldrich), α-carotene (≥98% Sigma-Aldrich), ethyl acetate UV/HPLC (Analitica) and acetonitrile UV/HPLC (Merck) were used.

For the *Artemia salina* assay, artificial sea water, brine shrimp eggs Maramar^®^, absolute methyl alcohol (Sigma-Aldrich) and potassium dichromate (Sigma-Aldrich) was used. For the MTT assay, cells of human colon carcinoma cell lines (T84) were purchased from the Institute of Molecular Medicine, University of Texas Health Science Center (one year before the onset of the experiments (with mycoplasma tests conducted)), medium DMEM-F12 (Sigma-Aldrich), fetal bovine serum (Gibco), penicillin antibiotic 50 UI.mL^-1^ (Gibco), streptomycin 50 μg.mL^-1^ (Gibco) and triton X-100 (Proquímios) were used.

For the comet assay, the following reagents were used: hydrochloric acid (CRQ), low melting point agarose (Agargen), standard agarose (Agargen), absolute ethanol (CRQ), ethidium bromide (Ludwig-Biotec), cyclophosphamide (Sigma-Aldrich) ethylenediaminetetracetic acid (Proquímios), heparin (Critália), potassium chloride (Vetec), monobasic anhydrous potassium phosphate (Scientific Exodus), pH kits (Impex), dibasic sodium phosphate (Dynamics), sodium chloride (Impex), sodium hydroxide (Vetec), saline (Arboretum), tris (Vetec) and triton X-100 (Proquímios).

For micronucleus test, hydrochloric acid (CRQ), absolute ethanol (CRQ), absolute methanol (Sigma-Aldrich), anhydrous monobasic potassium phosphate (Ex Scientific), dibasic sodium phosphate (dynamics), cyclophosphamide (Sigma- Aldrich), Giemsa (Laborclin), sodium hydroxide (Vetec), saline (Arboretum) and fetal bovine serum (Laborclin) were used. In order to prepare the oil used in the gavage administration, Tween^®^ 80 and saline (Arboretum) were used. For the euthanasia procedure, the anaesthetic Isoflurane (Cristalia) was applied.

### Carotenoids characterization: *High performance liquid chromatography*

The sample was analyzed using an analytical high performance liquid chromatography (HPLC) (LC-6AD, Shimadzu, Kyoto, Japan) system with a binary solvent. A photodiode array detector (PAD) was monitored at *λ * = 200–800 nm and column ODS HYPERSIL (C-18, 150 mm length x 4,6 mm inside diameter, particle size, 5 μm Thermo Electron Corporation). The elution was carried out using 90% acetonitrile, 10% ethyl acetate, in 15 min 50% acetonitrile, 50% ethyl acetate, in 25 min returning to the initial condition. The flow rate and injected volume were 0.7 mL min^-1^ and 20 μL, respectively. All chromatographic analyses were performed at 22°C. The content estimation of the compounds in the *A*. *phalerata* oil was performed by external calibration employing HPLC. A linear least-square regression of the peak areas as a function of the concentrations was performed to determine the correlation coefficients. The equation parameters (slope and intercept) of the standard curve were used to obtain the concentration values for the samples.

### Artemia salina assay

The lethality of APMO was evaluated using the brine shrimp lethality test (*Artemia salina*). The procedure was performed in accordance with Martin’s methodology [24]. Standard and sample solutions of the APMO (0.5–50 mg.mL^-1^ concentration) were prepared in methanol. After the solvent evaporation, 10 newborn *Artemia salina* nauplii were placed in test tubes containing 5 mL of artificial seawater. The assays were repeated four times for each concentration. Plates were observed after 24 hours of incubation using a Research Stereomicroscope System (Olympus SZX9) and the survival rate (%) was logged.

### MTT cytotoxicity assay

MTT (3-(4,5-dimethizzol-zyl)-2,5-diphenyltetrazolium bromide) assay was performed in accordance with Quassinti’s methodology [25] with some modifications. The T84 cells were incubated with DMEM-F12 with 10% fetal bovine serum in 96-well plate (well volume: 0.34 ml; growth surface: 0,31cm 2, inner diameter: 6.4 mm, dimensions (mm): 128 x 85 x 22) in a humidified incubator with 5% CO_2_ at 37°C for 72 hours until reaching 80–100% confluence. The cells were cultured in accordance with the ATCC protocol. Different concentrations of the APMO were prepared (1, 2.5, 5, 10 and 20 mg.mL^-1^) and incubated for 1, 24 and 48 hours. The medium was replaced by serum-free medium and 10 uL (5 mg.mL^-1^) of the MTT volume was added to it. Then, plates were incubated for 1–3 hours at 37°C (until reaching purple coloration), the medium removed and replaced by the solution of isopropyl alcohol/HCl 0.04 M. The mixture was stirred vigorously in a shaker until the purple color was homogeneous. The optical density was measured by a microplate reader (SpectraMax-M2^*e*^ *–*software SPECTRAmax M2e ROM versão 2.1) at 570 nm. Triton (1%) was used as positive control for cell viability.

### Animals and exposures

This study was carried out using 30 male and 30 female *Wistar* rats (*Rattus norvegicus*). The animals were at 8–10 weeks old, weighing between 235–243 g for females and 413–434 g for males. The animals obtained from the State University of Maringa were housed in polypropylene rodent cages under controlled temperature (23°C), humidity (40–60%) and 12h light/dark cycle with *ad libitum* access to water and standard commercial feed.

The rats were divided into six experimental groups of ten animals each (five males and five females). The APMO was diluted in saline + Tween 80^®^ and administered by gavage, in doses of 125, 250, 500 or 1000 mg.kg^-1^ body weight, daily for 28 consecutive days. The doses were chosen based on our subchronic toxicity studies in rats, and following the dose limit recommended by the OECD [26] for subacute treatments in toxicology assays. The negative control group received saline + Tween 80^®^. The positive control group received an intraperitoneal injection of cyclophosphamide of 20 mg.kg^-1^ body weight. The animals were observed daily during the whole experiment. Signs related to animal health and welfare were evaluated according to the Hippocratic screening, assessment of body weight, feed and water intake. If any animal presented any signs that would compromise its welfare, it would be euthanized in order to prevent suffering.

This study was carried out in strict accordance with the recommendations in the Guide for the Care and Use of Laboratory Animals of the National Institutes of Health. All experiments were approved by the Ethics Committee on Animal Research of the Federal University of Grande Dourados, under the permit number 21/2015. The animals used in this study were euthanized with inhalational anesthetic (isoflurane) in gas chamber followed by exsanguination.

### Comet assay

The comet assay (Single Cell Gel Electrophoresis—SCGE) was performed according to the protocol described by the OECD [18] with some modifications. Blood samples from all animals were obtained by caudal puncture with a needle soaked in Heparin and 40 uL of the blood was transferred to a micro-tube containing 120 uL of low melting point agarose (1.5%) at 37°C. The mixture was homogenized and transferred into pre-coated slides with 5% standard agarose. Two slides were prepared per animal. The slides were covered with coverslips and maintained in the dark at 3°C for 20 minutes.

After the waiting time, the coverslips were removed and the slides were immersed in lysis solution [89 mL stock lysis solution (2.5 M NaCl, 100 mM EDTA, 10 mM Tris, pH 10.0), and 89 mL distilled water], 1 mL Triton X-100, and 10 mL dimethyl sulfoxide for 1 hour at 3°C in the dark. After 1 hour, the slides were placed into the electrophoresis unit (Loccus) containing buffer solution at pH > 13 (300 Mm NaOH and 1 mM EDTA, prepared from a stock solution of 10 N NaOH and 200 mM EDTA, pH 10.0) at 4°C for 20 min in the dark, in order to allow the denaturation of the DNA.

The electrophoresis step was performed at 4°C for 20 min at 300 mA and 25 V, protected from light. After the run, the slides were submerged in neutralization buffer using 0.4 M Tris-HCl at pH 7.5 for three cycles of 5 min each, dried, fixed with 100% ethanol for 10 min, and stored for later analysis. Finally, the slides were stained with ethidium bromide (20 mg.mL^-1^) and covered with a coverslip. All material was analyzed by fluorescence microscopy (Nikon—H550S—40X magnification), equipped with an excitation filter (420–490 nm) and a barrier filter (520 nm).

Only individual nuclei were analyzed and the extent of damage in DNA was assessed by examining 100 cells per animal. The extent of DNA which migrated during electrophoresis and the migration distance reflects the amount and size of the DNA fragments. The comet findings were classified as follows: class 0—no damage; class 1—comet tail shorter than the diameter of the nucleoid; class 2—comet tail once or twice the diameter of the nucleoid and class 3—comet tail greater than twice the size of the nucleoid. Comets with a turbid aspect or a head that was too small were excluded from the analyses, as they could represent dead cells [27].

The damage index (DI) and damage frequency (DF) were calculated based on the readings. The DI was calculated by multiplying the number of cells damaged by the value assigned to this damage. Therefore, this value ranges from 0 (no damage: 0 x 100 cells) to 300 (maximum damage: 3 x 100 cells). DF was calculated by summing the number of damaged cells (class 1, 2 or 3) and ranged from 0 (no damage) to 100 (maximum damage).

### Micronucleus assay

The assay was performed following the protocol recommended by the OECD [19] with some modifications. The same animals used in the comet assay were also used in this test. The femoral bone marrow was removed, washed using 1 mL of bovine fetal serum and centrifuged (Spinlab—SL-5AM) for 5 minutes at 1000 rpm. The supernate was discarded and the pellets were used to make smears on slides. The slides were coded for blind analysis, fixed with methanol for 10 minutes and stained with Giemsa for 15 minutes. For the analysis of cells, 2000 polychromatic erythrocytes (PCE) per animal were evaluated for the presence of micronuclei in order to assess the clastogenic capacity of the APMO. The average number of micronucleated polychromatic erythrocytes (MNPCEs) per individual was used as the experimental unit and the standard deviation was based on the differences among animals of the same group.

To detect any cytotoxic effects, the relationship between PCE/NCE (polychromatic erythrocytes/normochromatic erythrocytes) was determined by analyzing 200 random erythrocytes per animal. The cells were analyzed by light microscope (Olympus—CX41) under magnification of 1000x.

### Statistical Analysis

The results were expressed as mean ± SEM. The differences between groups were determined by analysis of variance (one-way ANOVA) followed by Tukey’s test. P-values less than 0.05 were considered significant.

## Results and Discussion

### Chemical composition

The characterization of carotenoid by HPLC of APMO showed two major compounds (Table 1). The main compounds found in the oil are β-carotene and α-carotene, in which β-carotene is the major one. Due to the high concentration of the carotenoids, the results indicate that the oil will exhibit activities and act as a potential preventer of chronic diseases [1, 22]. Previous pharmacological studies performed with fruit pulp oils from the Cerrado with high levels of carotenoids, have shown great anti-edematogenicpotential in rats [7, 13] Quantitative values for β-carotene were evaluated in other fruits from the Cerrado, such as the *Caryocar brasiliense* Camb. (42.4 μg/g), *Annona crassiflora* Mart. (19.7 μg/g), *Eugenia dysenterica* DC. (3.96 μg/g), *Hymenaea stigonocarpa* Mart. (3.96 μg/g) e *Hancornia speciosa* Gomes (0.6 μg/g) [28].

**Table 1:** Characterization of the α-carotene and β-carotene values by chromatographic analysis in *Attalea phalerata* Mart. ex Spreng. oil (APMO).

	Standards	
	α carotene	β carotene
Retention time (min)	17.35	18.51
Linear Range (μg)	3–12	0.30–2
Intercept(a)	3995.30	4314.30
Slope (b)	4009.30	16411.00
Determination coefficient (R^2^)	0.993	0.991
Carotenoids concentration (μg/g)	10.93	61.72

Linear regression, formula: y = a + bx, where y = ratio of peak areas; x = concentration (μg); a = intercept and b = slope.

### Artemia salina assay

**Fig 1:**
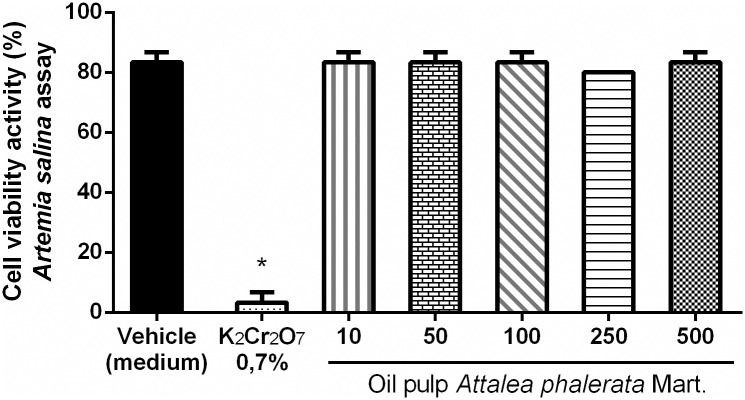
Effects of the APMO on *Artemia salina* assay. *Artemia salina* nauplii were treated with 10, 50, 100, 250 and 500 μg.mL^-1^ of oil or vehicle (medium) for 24 h. Results are presented as mean + SEM. n = 4; One-way ANOVA followed by Tukey´s test; *p<0.05 compared to vehicle (medium) and experimental group (10, 50, 100, 250 and 500 μg.mL^-1^ of oil).

The results of the brine shrimp nauplii survival rates were not statistically different (p <0.05) in all doses of the APMO when compared to the negative control group. As expected, the experimental groups (10, 50, 100, 250, and 500 μg.mL^-1^ of oil) and the negative control group presented statistical differences when compared to the positive control group (K_2_Cr_2_O_7_ 0.7%). These results demonstrate the low cytotoxicity of the APMO in microcrustaceans (Fig 1).

### MTT assay

**Fig 2:**
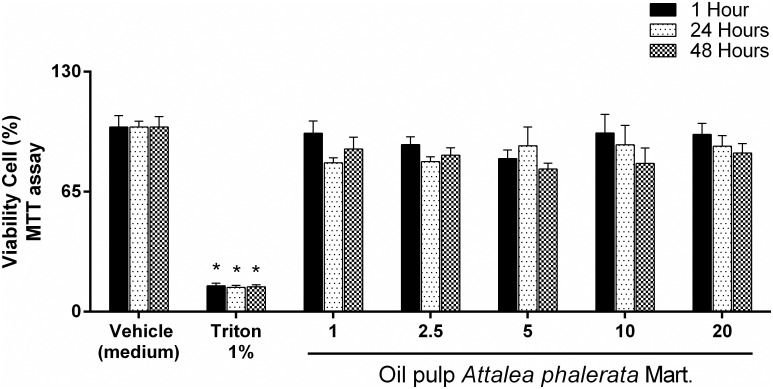
Effects of the APMO on cell metabolic activity by MTT assay. T84 cells were treated with 1, 2.5, 5, 10 and 1 mg.mL^-1^ of oil or vehicle (medium) for 1h, 24h and 48h. Results are presented as mean + SEM. n = 8; One-way ANOVA followed by Tukey´s test; *p<0.05 compared to vehicle (medium) and experimental group (1, 2.5, 5, 10 and 1 mg.mL^-1^ of oil).

The MTT results (Fig 2) showed the absence of cytotoxicity of the APMO at doses of 1, 2.5, 5, 10 and 20 mg/mL^-1^ (dose and time dependent manner) where the experimental doses did not differ statistically (p <0.05) when compared to the negative control group (medium). However, the experimental groups and the negative control group showed statistical differences when compared to the positive control group (Triton 1%).

### Comet and Micronuclei assays

Comet and micronuclei assays are effective tests to determine the cytotoxicity, genotoxicity and clastogenicity. The *in vivo* release from mammalian cells under alkaline conditions in the comet assay was used in our study, according to the OECD [19]. This test provides a broad-spectrum detection of the levels of DNA damage, such as DNA denaturation and detection of alkali-labile sites [3–5]. Cells obtained by caudal puncture from rats were analyzed. The results obtained with the comet assay (Table 2) show DNA damage (according to the size of the tail) in peripheral blood leukocytes (harvested after daily treatment for four weeks). Cell viability for the negative control group (saline + Tween 80) was higher compared to the positive control group (cyclophosphamide 20 mg.kg^-1^), confirming the low genotoxicity rate in healthy rats. As expected, cyclophosphamide significantly increased the DNA leukocyte migration (p <0.05) when compared to the negative control group. At doses of 125, 250, 500 and 1000 mg.kg^-1^ of the APMO, there was no significant increase in DNA breaks (p <0.05) when compared to the negative control group. Most cells exposed to four concentrations of test compound were examined in slides and showed no DNA damage (Class 0) followed by a few class 1 DNA damage events.

**Table 2:** Effect of the APMO in exposed rats as measured by the comet assay. The groups were treated with 125, 250, 500 and 1000 mg.kg^-1^ of the *Attalea phalerata* Mart. ex Spreng. oil (APMO) daily for 28 consecutive days (four weeks) and the negative control group received the vehicle (saline + Tween 80^®^). The positive control group received intraperitoneal injection of cyclophosphamide at 20 mg.kg^-1^.

Groups	Damage frequency	Classes of damage				Damage index
		0	1	2	3	
Female						
Negative Control	6.20 ± 0.73^a^	93.80 ± 0.73	6.20 ± 0.73	0.00 ± 0.00	0.00 ± 0.00	6.20 ± 0.73^a^
Cyclophosphamide	85.40 ± 1.75^b^	14.60 ± 1.75	41.20 ± 1.07	38.80 ± 1.68	5.40 ± 1.83	135.00 ± 5.07^b^
125 mg.kg^-1^ of APMO	8.40 ± 2.01^a^	91.60 ± 2.01	8.00 ± 2.07	0.40 ± 0.24	0.00 ± 0.00	8.80 ± 1.98^a^
250 mg.kg^-1^ of APMO	8.60 ± 2.80^a^	89.25 ± 2.32	10.75 ± 2.32	0.00 ± 0.00	0.00 ± 0.00	8.60 ± 2.80^a^
500 mg.kg^-1^ of APMO	6.20 ± 0.58^a^	93.80 ± 0.58	5.80 ± 0.66	0.40 ± 0.24	0.00 ± 0.00	6.60 ± 0.60^a^
1000 mg.kg^-1^ of APMO	7.80 ± 2.18^a^	90.25 ± 1.25	8.75 ± 1.11	0.75 ± 0.48	0.25 ± 0.25	8.80 ± 2.56^a^
Male						
Negative Control	6.60 ± 1.21^a^	93.40 ± 1.21	6.00 ± 1.38	0.60 ± 0.40	0.00 ± 0.00	7.20 ± 1.16^a^
Cyclophosphamide	89.60 ± 1.50^b^	10.40 ± 1.50	41.80 ± 1.69	40.60 ± 1.50	7.20 ± 1.98	144.60 ± 2.87^b^
125 mg.kg^-1^ of APMO	11.80 ± 0.97^a^	88.20 ± 0.97	9.80 ± 1.16	2.00 ± 0.71	0.00 ± 0.00	13.80 ± 1.24^a^
250 mg.kg^-1^ of APMO	13.20 ± 3.73^a^	87.00 ± 3.79	12.80 ± 3.83	0.40 ± 0.40	0.00 ± 0.00	13.60 ± 3.67^a^
500 mg.kg^-1^ of APMO	9.00 ± 1.52^a^	91.00 ± 1.52	8.80 ± 1.50	0.20 ± 0.20	0.00 ± 0.00	9.20 ± 1.56^a^
1000 mg.kg^-1^ of APMO	12.60 ± 2.52^a^	87.40 ± 2.52	11.40 ± 2.54	1.00 ± 0.45	0.20 ± 0.20	14.00 ± 2.70^a^

Negative control—saline solution + Tween 80^®^; Cyclophosphamide– 20 mg.kg^-1^ip.; *Attalea phalerata* Mart. oil at doses of 125, 250, 500 e 1000 mg.kg^-1^ by gavage. Different letters (a and b) indicate statistically significant differences (p< 0.05) of positive group (cyclophosphamide) between negative control and experimental group (doses 125, 250, 500 e 1000 mg.kg^-1^); ANOVA/Tukey

**Fig 3:**
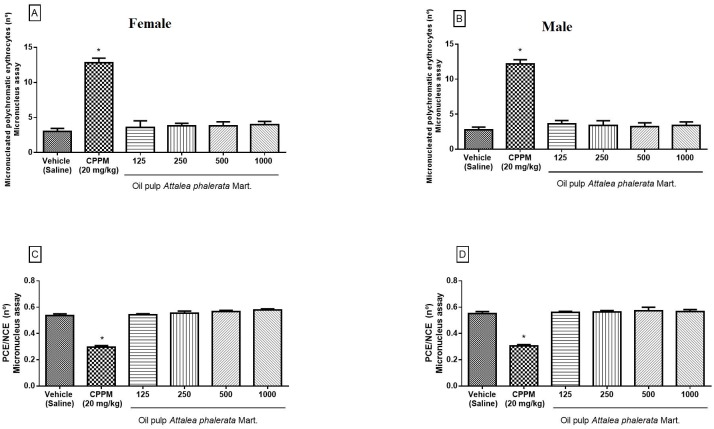
Effect of the APMO on Micronucleated Polychromatic Erythrocytes (Fig 3A and 3B) and the ratio between Polychromatic Erythrocytes/Normochromatic Erythrocytes—PCE/NCE (Fig 3C and 3D) micronucleus assay. Groups of female (Fig 3A and 3C) and male (Fig 3B and 3D) rats were treated with 125, 250, 500 and 1000 mg.kg^-1^ of the APMO daily for 28 consecutive days (four weeks) and the negative control group received the vehicle (saline + Tween 80^®^). The positive control group received intraperitoneal injection of cyclophosphamide (CPPM) 20mg.kg^-1^. Results are presented as mean + SEM; One-way ANOVA followed by Tukey´s test; *p<0.05 compared to vehicle (saline) and experimental group (treated with 125, 250, 500 and 1000 mg.kg^-1^ of the oil).

The in vivo version of the micronucleus test in mammalian cells was performed in our study [19] and enabled evaluation of the presence or absence of damage caused by the chemical compounds of the APMO on chromosomes and/or mitotic apparatus of erythroblasts [6, 19, 29]. This *in vivo* test is the main test which assess the genetic toxicology to provide cytotoxic and mutagenic results and is highly recommended by global regulators. In this study, cells obtained from the marrow bone of the animals were analyzed after daily treatment with the APMO, for four weeks, at four different doses, as showed in Fig 3.

There was no statistical difference (p <0.05) in the frequency of micronuclei in polychromatic erythrocytes (MNPCEs) among the negative control group (saline + Tween 80) and the groups treated with four doses of the APMO, indicating no clastogenic effects of this oil. As expected, the treated animals in the positive control group (cyclophosphamide 20 mg.kg^-1^) showed large numbers of micronucleated polychromatic erythrocytes in bone marrow cells when compared to the negative control and to the experimental groups (p <0.05). The estimated ratio of PCE/NCE in bone marrow preparations showed no statistical significant changes in hematopoiesis after the administration of the APMO, indicating no cytotoxic effects from the use of *A*. *phalerata* oil.

No published studies involving the evaluation of cytotoxic, genotoxic and clastogenic potential of *A*. *phalerata* oil were found. Thus, the results corroborate with studies conducted with Cerrado fruits, in which the antigenotoxic/genotoxic and antimutagenic/mutagenic activities were evaluated [30, 31], with satisfactory results for both parameters and driving interest in evaluating the possible antigenotoxic and antimutagenic activities of the APMO in future studies.

## Conclusions

The results obtained in the micronucleus test are consistent with those observed by the comet assay, as well as *Artemia salina* and MTT assays. In conclusion, the results of this study demonstrate that the *A*. *phalerata* pulp oil has no cytotoxicity, genotoxicity and clastogenicity at doses tested in the experimental models used. The results demonstrated the safe use of the fruit pulp oil in folk medicine. In an ethnopharmacological context, the trials are considered important prerequisites for the identification of genetic diseases. Although the genotoxicity is not a direct measure of carcinogenicity, it is often used as an indicator for cancer, since the tests measure an initial or intermediate event in tumorigenesis.
